# A Prodrug Approach to the Use of Coumarins as Potential Therapeutics for Superficial Mycoses

**DOI:** 10.1371/journal.pone.0080760

**Published:** 2013-11-18

**Authors:** Derry K. Mercer, Jennifer Robertson, Kristine Wright, Lorna Miller, Shane Smith, Colin S. Stewart, Deborah A. O′Neil

**Affiliations:** NovaBiotics Ltd, Craibstone, Bucksburn, Aberdeen, United Kingdom; University of Wisconsin Medical School, United States of America

## Abstract

Superficial mycoses are fungal infections of the outer layers of the skin, hair and nails that affect 20–25% of the world's population, with increasing incidence. Treatment of superficial mycoses, predominantly caused by dermatophytes, is by topical and/or oral regimens. New therapeutic options with improved efficacy and/or safety profiles are desirable. There is renewed interest in natural product-based antimicrobials as alternatives to conventional treatments, including the treatment of superficial mycoses. We investigated the potential of coumarins as dermatophyte-specific antifungal agents and describe for the first time their potential utility as topical antifungals for superficial mycoses using a prodrug approach. Here we demonstrate that an inactive coumarin glycone, esculin, is hydrolysed to the antifungal coumarin aglycone, esculetin by dermatophytes. Esculin is hydrolysed to esculetin β-glucosidases. We demonstrate that β-glucosidases are produced by dermatophytes as well as members of the dermal microbiota, and that this activity is sufficient to hydrolyse esculin to esculetin with concomitant antifungal activity. A β-glucosidase inhibitor (conduritol B epoxide), inhibited antifungal activity by preventing esculin hydrolysis. Esculin demonstrates good aqueous solubility (<6 g/l) and could be readily formulated and delivered topically as an inactive prodrug in a water-based gel or cream. This work demonstrates proof-of-principle for a therapeutic application of glycosylated coumarins as inactive prodrugs that could be converted to an active antifungal in situ. It is anticipated that this approach will be applicable to other coumarin glycones.

## Introduction

Superficial mycoses are fungal infections of the outer layers of the skin, hair and nails [1] and are the most common fungal infections [2]. The most common causative microbes are dermatophytes (*Trichophyton* spp., *Microsporum* spp. and *Epidermophyton* spp.) [2]. Other fungi, including *Scopulariopsis brevicaulis, Malassezia* spp., *Alternaria* spp., *Fusarium* spp. and *Candida* spp. can cause or be isolated from superficial mycoses [1].

Treatment of superficial mycoses is generally by topical therapeutics in the first instance, including azoles, polyenes and allylamines [3]. Topical treatments have advantages over systemic treatments; fewer and less severe adverse effects, improved patient compliance and lower overall cost of treatment [3]–[6]. Ideally, a new topical antifungal would be fungicidal rather than fungistatic so treatment cycles can be shorter, higher cure rates attained, the chance of resistance reduced and the chance of relapse lowered [4]. The use of water-soluble antifungal agents would be preferable to facilitate ease of formulation and ameliorate potential irritant effects of solvents.

Coumarins are plant-derived polyphenolic compounds, best known therapeutically for their anti-inflammatory, anti-thrombotic (e. g. warfarin) and vasodilatory properties [7]. Selected coumarins possess antimicrobial properties [8]–[11]. Coumarins are predominantly poorly water-soluble, but coumarin glycosides are significantly more water-soluble, albeit with reduced/no antimicrobial activity, and are readily available or can be synthesized simply [12]. Coumarin glycosides are hydrolysed to the active coumarin aglycone by β–glucosidases; hydrolysis of esculin to esculetin has been suggested to reduce the survival of certain pathogens in anaerobic gut habitats [8].

Prodrugs are pharmacological substances administered in an inactive (or significantly less active) form, that once administered, are converted into the pharmacologically active agent by metabolic or physico-chemical transformation [13]. A prodrug is often used to improve ADME properties, increase selectivity for the intended target, facilitate ease of formulation, or deliver a cytotoxic agent, in a non-toxic form, to a specific site where it is metabolised into the active form [14]. The prodrug approach, relatively common in cancer therapeutics, has received less attention in the field of antimicrobials, but is used with colistin methanesulfonate [15] and fosfluconazole [16]. Many antifungal molecules demonstrate poor aqueous solubility, so preparations for topical application often require solvents that can have irritant side-effects [5], [6]. Delivery of water-soluble prodrugs that are converted to the active form at the site of infection would, therefore, be a desirable property.

In this manuscript we demonstrate the proof-of-principle that an inactive water-soluble coumarin glycoside (esculin) can be converted to the active antifungal coumarin (esculetin) by β–glucosidase activity, an enzyme produced by the target dermatophytes. The use of β–glucosidase inhibitors ameliorates antifungal activity. We also demonstrate that members of the dermal commensal microbiota can also hydrolyse esculin to esculetin and that these concentrations are not sufficient to kill selected members of the dermal microbiota. The advantages of this approach are that the inactive form of the drug can be delivered in an aqueous vehicle and it is only converted to the active form when exposed to the target dermatophytes or other members of the skin microbiota. The specificity of esculetin means that at the concentrations intended to be applied, there should be little/no impact on the skin microbiota, but the infecting dermatophytes should be killed. It should be possible to apply this mechanism to more active antimicrobial coumarins than esculetin using this novel targeted prodrug approach.

## Results and Discussion

### Determination of the MIC and MFC of selected coumarin glycones and aglycones

The antifungal effect (MIC_100_) of selected coumarins against fungi isolated from human dermal infections were determined (Table 1). The coumarin glycosides, esculin and fraxin were inactive against all 107 isolates of fungi tested (MIC_100_>3400 µg/ml esculin, MIC_100_>3700 µg/ml fraxin, MIC_100_>3400 µg/ml). The coumarin aglycones esculetin, daphnetin and fraxetin demonstrated antifungal activity against the dermatophytes tested at concentrations of 11.13–416.34 µg/ml (84 strains). No trends were observed that indicated one coumarin aglycone demonstrated significantly better antifungal activity than any other. Esculetin and daphnetin differ only in the position of their hydroxyl groups (6,7-dihydroxycoumarin and 7,8-dihydroxycoumarin, respectively), whereas fraxetin is identical to daphnetin, but with the addition of a 6-methoxy group. Non-dermatophyte dermal isolates tested; *Fusarium* spp. (n = 11), *S. brevicaulis* (n = 7) and *A. fumigatus* (n = 1) were not sensitive to esculetin, daphnetin or fraxetin (MIC_100_>416.34 µg/ml). Esculetin was also ineffective against yeasts; *Candida albicans* SC5314, *C. krusei* ATCC6258 (cutaneous candidiasis) and *Malassezia furfur* DSMZ6170 (pityriasis versicolor) (MIC_100_>696.56 µg/ml). All bacteria tested for esculin hydrolysis were able to grow on 1 g/l esculin, irrespective of β–glucosidase activity, indicating that esculin and esculetin are unlikely to have adverse effects on commensal skin bacteria. Conventional antifungals used to treat superficial mycoses caused by dermatophytes include ketoconazole (topical), terbinafine (topical or oral), itraconazole (oral) tolnaftate (topical) and other azoles (both oral and topical). In general, the in vitro MIC values of these antifungals are lower than esculetin (0.001-≥64 µg/ml versus 11.13–416.34 µg/ml)[17]. However, when applied topically they are typically used at much higher concentrations; 2% ketoconazole (cream), 1% terbinafine (spray, cream, gel or cutaneous solution), 1% clotrimazole (cream) and 1% tolnaftate (cream) and these are similar to the intended concentration for esculin. Dermatophytes in superficial mycoses exist as both spores and hyphae [1]. Antifungal susceptibility testing using the method used primarily uses a concentrated spore suspension and it is well known that spores are more resistant to antifungals than hyphae. Therefore, esculetin, daphnetin and fraxetin should kill dermatophytes irrespective of their physiological state. Therefore, esculetin, daphnetin and fraxetin appear to be dermatophyte-specific antifungals unlikely to affect the balance of the commensal dermal microbiota if used as a treatment for superficial mycoses caused by dermatophytes. The fungicidal activity of esculetin was determined and MFCs indicate that esculetin is fungicidal against *T. interdigitale* NCPF0335 (174.18 µg/ml) and *T. rubrum* NCPF0118 (348.36 µg/ml). The MIC_100_ of esculetin was 50% lower than the fungicidal concentration against *T. rubrum* NCPF 0118 whereas the MIC and MFC versus *T. interdigitale* NCPF0335 were identical. Fungicidal activity is desirable, as this is likely to decrease the treatment time required to eliminate the pathogen. Interestingly, the active antifungal aglycones, esculetin, daphnetin and fraxetin, are dihydroxycoumarins, whereas singly hydroxylated coumarins, e. g. 4-hydroxycoumarin and 6-hydroxycoumarin, demonstrated no antifungal activity at concentrations up to 410.35 µg/ml (data not shown). In an earlier study, Johann, et al., [18] demonstrated that scoparone (6,7-dimethoxycoumarin) demonstrated antifungal activity (MIC_100_ = 250 µg/ml) against a *T. mentagrophytes* clinical isolate. It would therefore appear that the coumarin aglycones tested here are dermatophyte-specific antifungal agents, and require 2 hydroxyl groups for activity. However, coumarin aglycones are not water-soluble, without the use of a vehicle such as DMSO, at concentrations likely to be of use clinically. DMSO can promote absorption through the skin, which would be undesirable in this case [19].

**Table 1:** Minimal Inhibitory Concentration (MIC) of coumarin glycones and aglycones against fungi isolated from human superficial mycoses.

	Median MIC_100_ (µg/ml) (range)				
Genus & species	Esculin	Esculetin	Daphnetin	Fraxin	Fraxetin
*Trichophyton interdigitale* (n = 3)	>3402.8	178.14 (89.07–356.28)	89.07 (22.27–89.07)	>3703.1	104.09 (52.04–104.09)
*Trichophyton mentagrophytes* (n = 24)	>3402.8	178.14 (22.27–356.28)	89.07 (22.27–356.28)	>3703.1	104.09 (13.01–208.17)
*Trichophyton rubrum* (n = 50)	>3402.8	89.07 (11.14–356.28)	22.27 (11.13–356.28)	>3703.1	26.02 (13.01–416.34)
*Trichophyton soudanense* (n = 2)	>3402.8	22.27 (22.27–89.07)	22.27 (22.27–174.18)	>3703.1	26.02 (13.01–26.02)
*Trichophyton tonsurans* (n = 2)	>3402.8	89.07 (22.27–89.07)	22.27 (22.27)	>3703.1	26.02 (26.02–208.17)
*Microsporum* canis (n = 3)	>3402.8	178.14 (89.07–356.28)	22.27 (11.14–44.54)	>3703.1	26.02 (13.01–26.02)
*Fusarium* spp. (n = 11)	>3402.8	>356.28	>356.28	>3703.1	>416.34
*Aspergillus fumigatus* (n = 1)	>3402.8	>356.28	>356.28	>3703.1	>416.34
*Scopulariopsis brevicaulis* (n = 7)	>3402.8	>356.28	>356.28	>3703.1	>416.34

All results are presented as the median MIC values from at least triplicate experiments with triplicate samples within each experiment. In all cases, the standard deviation was within 10% of the mean. n = no. strains tested.

The mechanism of action of coumarins against fungi is not known and it is not clear why dermatophytes appear to be more susceptible than other filamentous fungi or yeasts isolated from dermal infections. The antibacterial effect of the aminocoumarin antibiotic novobiocin targets the GyrB sub-unit of bacterial DNA gyrase and blocks ATP-ase activity thereby inhibiting protein and nucleic acid synthesis [20], but in the case of novobiocin the carbamoyl group located on the novobiose sugar is responsible for significant antibacterial activity, and this moiety is not found on the coumarins tested here. Esculetin has been described as an inhibitor of tyrosinases and polyphenol oxidase, key enzymes involved in melanin formation [21], and this property could inhibit melanin formation by dermatophytes [22]. Melanins are known virulence determinants associated with decreased susceptibility to other antifungals, including amphotericin B and caspofungin [23], and this may relate to the mechanism of action of the coumarin aglycones tested.

Esculin was soluble in RPMI-1640 medium at 6806 µg/ml, whereas fraxin was soluble at 7406 µg/ml. Similarly, both compounds were soluble at the same concentration in an aqueous 65% (w/v) PEG14000 gel with a texture similar to many gels and creams used for application of topical medications. PEG14000 is commonly found in a number of topical cosmetics and medications [24]. Therefore, esculin was soluble in the PEG14,000 vehicle at concentrations 19.1–611.5-fold higher than the range of MIC_100_ of esculetin versus dermatophytes tested in this study (11.14–356.28 µg/ml). Fraxin was soluble in the PEG14,000 vehicle at concentrations 17.8–669.3-fold higher than the MIC_100_ of fraxetin versus dermatophytes tested in this study (13.01–416.34 µg/ml) (Table 1). It should therefore be possible to prepare coumarin glycones as suspensions at higher concentrations than as solutions as is common for a number of antimicrobial creams/gels. Alternatively, the use of excipients in the topical formulation may facilitate dissolution of esculin at higher concentrations. Whilst it is not possible to determine the amount of esculin converted to esculetin in superficial mycoses without in vivo experiments, it is not unreasonable to assume that enough could be converted to demonstrate an antifungal effect against dermatophytes based on the difference between solubility and MIC_100_. Therefore, we set out to examine the mechanism of conversion of esculin to esculetin and whether dermatophytes and/or other members of the dermal microbiota could hydrolyse esculin to esculetin.

Whilst it is possible that esculetin, daphnetin and fraxetin may have application as topical antifungal agents against superficial mycoses caused by dermatophytes, their lack of aqueous solubility may make formulation more problematic. It was therefore decided to test the possibility that the coumarin glycone, esculin, might function as a water-soluble prodrug for delivery of the antifungal, esculetin. In order to generate the antifungal aglycone, β-glucosidase activity at the site of infection would be essential. If that was the case, then other water-soluble coumarin glycosides (e. g. daphnin and fraxin) may have similar potential. The reason esculin was selected, rather than fraxin or daphnin, was that esculin is already an approved medicinal compound (Proctosedyl ointment/suppositories; and as a generic product Cinchocaine Hydrochloride/Hydrocortisone ointment), readily available at GMP-quality (US Pharmacopoeia) and therefore already has safety data to support its use, unlike daphnin or fraxin. Additionally, esculin and esculetin have been reported to have potent anti-oxidative and photo-protective effects [25]), which could provide additional benefit to an antimycotic cream. The prodrug approach adopted for esculin should be equally applicable to daphnin, fraxin and other antifungal coumarin glycosides.

### Hydrolysis of esculin to esculetin by β–glucosidase and antifungal activity

**Figure 1:**
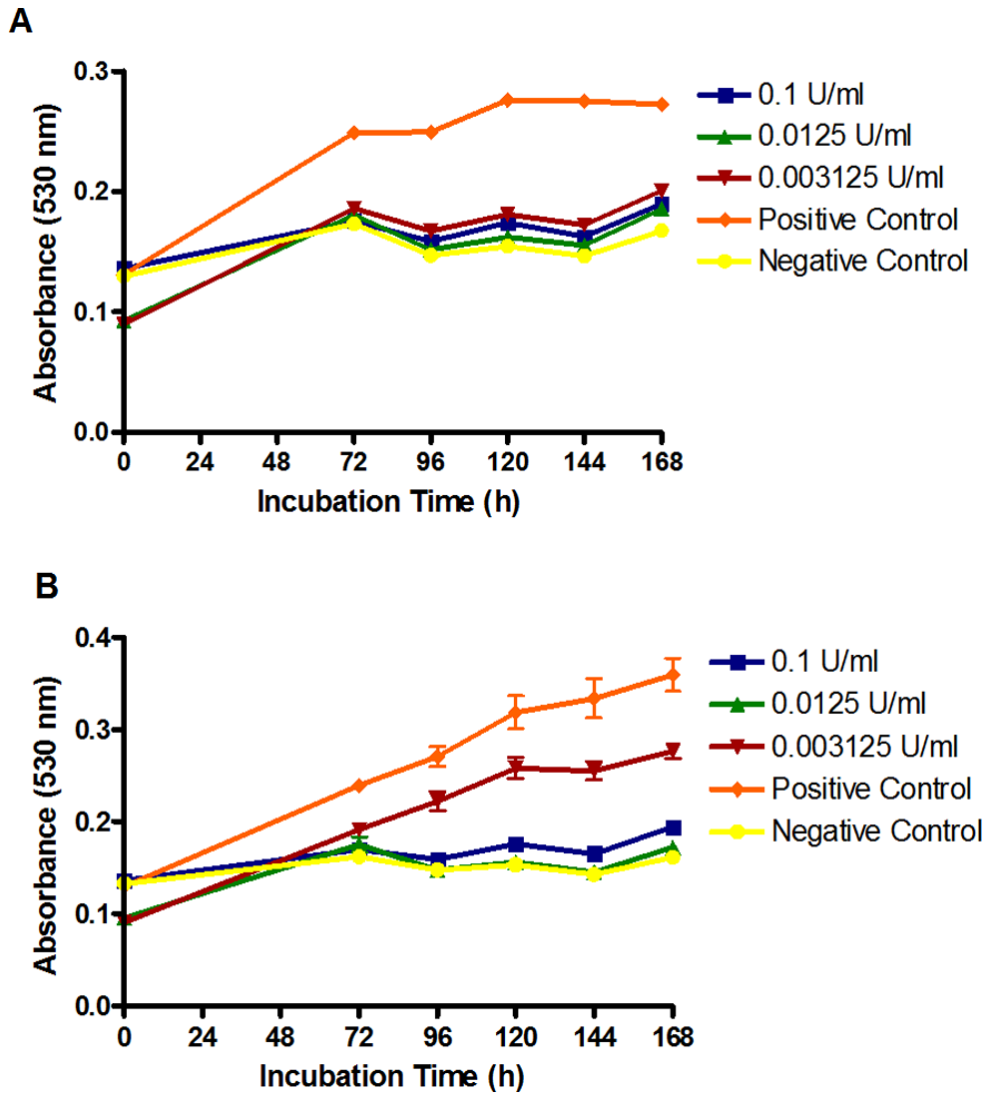
Determination of the amount of exogenous β-glucosidase required to exert an antifungal effect from esculin (3402.8 µg/ml). The negative control containing 348.36 µg/ml esculetin + 0.005 U/ml β-glucosidase demonstrated total kill for both strains. The positive control contains 0.005 U/ml β-glucosidase but no esculin and resulted in no growth inhibition. β-glucosidase at all concentrations tested demonstrated no antifungal activity. All results are representative of triplicate samples from triplicate experiments. Error bars represent the standard deviation of the mean.

Based on the hypothesis that esculin could be used as a prodrug to deliver esculetin to the site of fungal infection and that β–glucosidases can hydrolyse esculin to esculetin, or an antifungal product, it was necessary to confirm that the product of esculin hydrolysis by β–glucosidase activity was antifungal. Based on the reaction catalysed by β–glucosidases, the likely reaction products are esculetin and glucose. Therefore, MIC experiments were repeated, but with the addition of discrete amounts of almond β-glucosidase to incubations containing 3402.8 µg/ml esculin. In all cases, esculin (≤3402.8 µg/ml) or β-glucosidase (<0.5 U/ml) alone demonstrated no antifungal activity (data not shown). Addition of 0.005–0.063 U/ml almond β-glucosidase resulted in complete inhibition of growth of 8 *T. rubrum* and 2 *T. mentagrophytes* clinical isolates (data not shown). The concentrations of β–glucosidase used in this experiment may be higher than encountered by dermatophytes in superficial mycoses. Therefore, we determined the minimum amount of almond β-glucosidase activity required to kill *T. rubrum* NCPF0118 and *T. interdigitale* NCPF0335 in the presence of an excess of esculin (3402.8 µg/ml). As can be seen from Figure 1a, <0.003 U/ml of β–glucosidase is required to hydrolyse esculin and kill *T. rubrum* NCPF0118, whereas from Figure 1b, 0.003 – 0.006 U/ml β–glucosidase is required to hydrolyse esculin and kill *T. interdigitale* NCPF0335. Limited inhibition of *T. interdigitale* NCPF0335 growth is observed in the presence of 0.003 U/ml β–glucosidase. The amount of dermatophyte β–glucosidase required to generate sufficient esculetin is not known, but it is not unreasonable to hypothesize that similar active units of almond and dermatophyte β–glucosidase will be required. Therefore, the two *Trichophyton* spp. used above were tested for the production of extracellular β–glucosidase activity.

### β–glucosidase production by *T. rubrum* NCPF0118 and *T. interdigitale* NCPF0335

**Figure 2:**
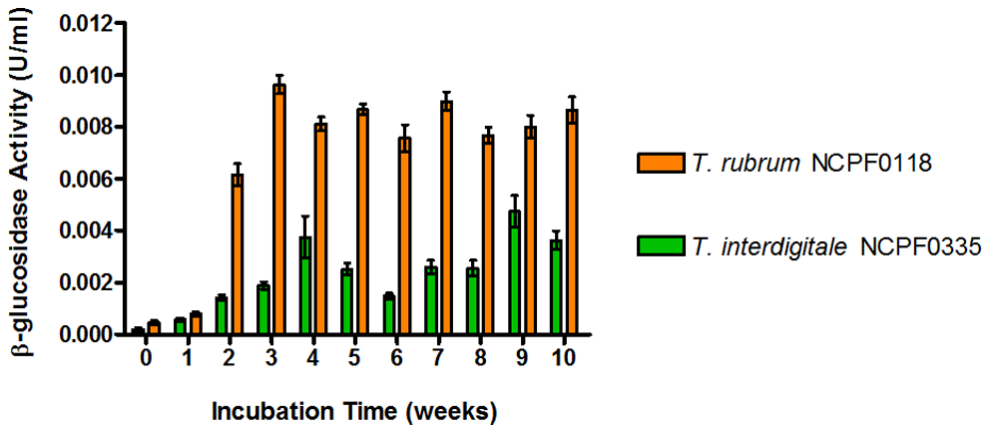
Determination of β-glucosidase activity by *T. rubrum* NCPF0118 and *T. interdigitale* NCPF0335 over a 10 week incubation period. β-glucosidase activity was determined from triplicate 100 µl cell pellets, and results are representative of triplicate experiments. Error bars represent the standard deviation of the mean.

Most microbial infections are acute in nature and are rapidly symptomatic shortly after infection, whereas superficial mycoses may be established for weeks or months before symptoms become apparent and treatment commences. We hypothesize that extracellular β-glucosidases produced by dermatophytes may accumulate at the site of infection and convert esculin to esculetin. Although it has been shown that dermatophytes produce β–glucosidases (Otsuka, et al, 1979; Papini & Mancianti, 1995), there have been no studies of esculin hydrolysis by dermatophytes. Additionally, there is no indication whether β–glucosidase production by dermatophytes is constitutive or inducible. β-glucosidase activity was detectable in culture supernatants of *T. rubrum* NCPF0118 and *T. interdigitale* NCPF0335 grown in static culture (Figure 2). *T. rubrum* NCPF0118 produced significant amounts of β-glucosidase activity after 3 weeks incubation, and the enzyme activity was maintained over the entire 10 week incubation period. *T. interdigitale* NCPF0335 produces significant (albeit less than *T. rubrum* NCPF0118) amounts of β-glucosidase activity after 4 weeks incubation, but for this strain, the enzyme activity appears to be biphasic. Based on the early production of β–glucosidases by both strains, it is likely that β–glucosidase production is constitutive.

In these experiments, only extracellular β–glucosidase activity was detected. However, by analogy with other fungi, autolysis seems likely to contribute to the release of β-glucosidases and other enzymes. Autolysis is an important adjunct to fungal development; the cell constituents released upon autolysis are assumed to provide nutrients which support fresh growth. In *Aspergillus nidulans* autolysis was triggered by gene products involved in the control of sporulation and growth (Emri, et al, 2005). These events have not been studied in dermatophytes, but may be important for understanding the potential for pro-drug processing of esculin in superficial mycoses. Both dermatophyte strains tested also produced intracellular β–glucosidases (data not shown). In the natural environment of the skin, microbial β-glucosidases could also be supplied by other members of the skin microbiota, including bacteria (Grice, et al, 2009).

### Antifungal activity of esculin breakdown products following incubation in the presence of esculin by conditioned supernatants from aged cultures of *T. rubrum* NCPF0118 and *T. interdigitale* NCPF0335

**Figure 3:**
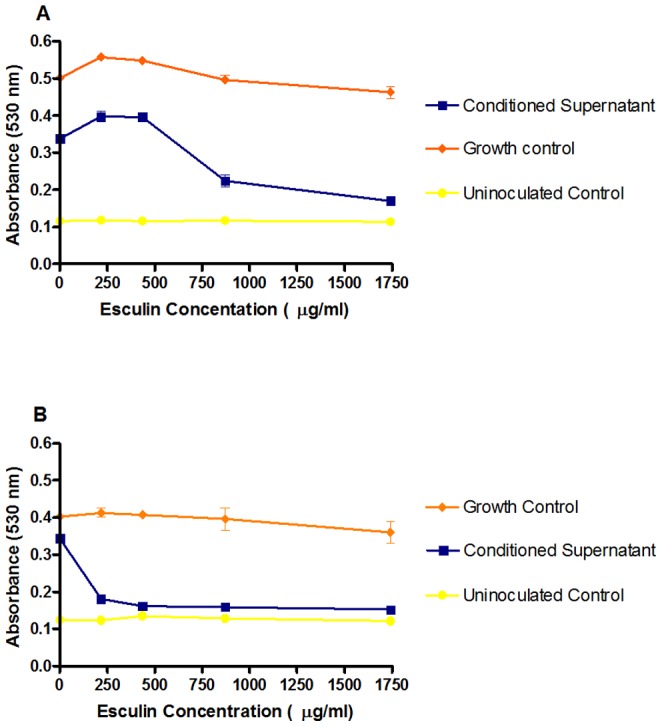
Analysis of the effect of 8 week old conditioned supernatants on the antifungal activity of esculin. Conditioned supernatants were used against *T. rubrum* NCPF0118 (A) and *T. interdigitale* NCPF0335 (B) grown for 7 d at 30°C. All results are representative of triplicate samples from triplicate experiments. Error bars represent the standard deviation of the mean.

To determine whether antifungal esculin breakdown products were generated in the presence of conditioned supernatants from older cultures which will probably be more representative of dermatophytes in nutrient-limited established infections compared with the young cultures typically employed to provide inocula for MIC testing. *T. rubrum* NCPF0118 and *T. interdigitale* NCPF0335 were grown for 2 or 8 weeks on RPMI-1640 medium containing 348.36 µg/ml esculin. Esculin was included as a potential inducer of β-glucosidase production. Filter-sterilized supernatants from these incubations (conditioned supernatants) were prepared after 2 and 8 weeks incubation and used to supplement (40% v/v) fresh incubations of the same strains grown on RPMI-1640 medium containing 0–3402.8 µg/ml esculin. No antifungal effects towards either strain were observed in the presence of 2 week conditioned supernatants +/− esculin (data not shown). In the presence of 8 week old conditioned supernatants, significant antifungal activity was observed versus both *T. rubrum* NCPF0118 (Figure 3a) and *T. interdigitale* NCPF0335 (Figure 3b). Complete hydrolysis of esculin by β–glucosidase should generate esculetin in a 1∶1 ratio. Growth of *T. interdigitale* NCPF0335 (esculetin MIC_100_ = 174.18 µg/ml) was significantly inhibited in the presence of 217.68 µg/ml esculin, indicating >80% conversion of esculin to escueltin. Growth of *T. rubrum* NCPF0118 (esculetin MIC_100_ = 174.18 µg/ml) was significantly inhibited in the presence of 870.70 µg/ml esculin, indicating significantly less efficient esculin hydrolysis by this strain. Incubations with 8 week old conditioned supernatants, but without esculin, demonstrated no antifungal activity, indicating that esculin and its breakdown product/s are essential for antifungal activity. This result demonstrates that dermatophytes can produce sufficient β-glucosidase to hydrolyse esculin to esculetin and exert an antifungal effect.

### Inhibition of *T. rubrum* NCPF0118 β-glucosidase activity and antifungal efficacy

**Figure 4:**
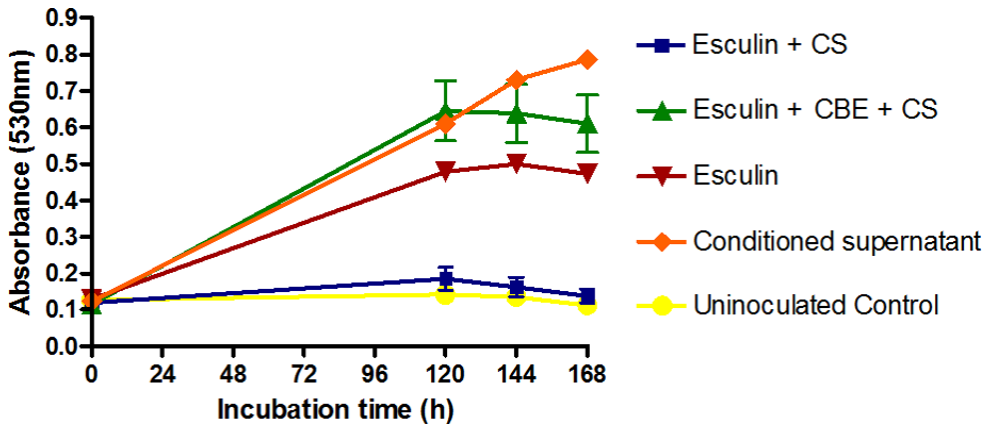
Effect of the β–glucosidase inhibitor Conduritol B epoxide (CBE; 40 µg/ml) on growth inhibition of *T. rubrum* NCPF0118. Esculin concentration = 1704.1 µg/ml; Esculetin concentration = 696.72 µg/ml. CS – Conditioned supernatant. Conduritol B epoxide (CBE) Concentration = 40 µg/ml. The esculetin control inhibited growth for the entire 168 h. All results are representative of triplicate samples from triplicate experiments. Error bars represent the standard deviation of the mean.

To confirm that β–glucosidases produced by *T. rubrum* NCPF0118 are responsible for esculin hydrolysis and antifungal activity, the β–glucosidase inhibitor conduritol B epoxide (CBE; 40 µg/ml) [26] was added to cultures of *T. rubrum* NCPF0118 growing on RPMI-1640 medium supplemented with 1701.4 mg/ml esculin and 8 week old conditioned supernatants. The results (Figure 4) confirm that the antifungal effect of esculin and conditioned supernatant is ameliorated by the addition of CBE. This indicates that the hydrolysis of esculin by conditioned supernatants is a result β–glucosidase activity. Addition of 40 µg/ml CBE to cultures of *T. rubrum* NCPF0118, in the presence or absence of esculin, demonstrated no inhibition of growth (data not shown).

### Esculin hydrolysis by bacteria typical of the skin microbiota

In addition to dermatophytes, β-glucosidases on the skin could also be supplied by other members of the skin microbiota, including bacteria [27] and skin cells themselves. Most staphylococci (the most common genus of the skin microbiota) and some *Corynebacterium* spp. are capable of hydrolysing esculin to esculetin [28]. The number of dermal bacteria capable of hydrolysing esculin to esculetin has not been accurately determined. Some fungi and yeasts that are members of the skin microbiota also produce β-glucosidases including dermatophytes [29] and *Malassezia sympodialis* [30]. Additionally, dermal fibroblasts and keratinocytes produce β-glucosidases (β-glucocerebrosidase [31], [32] and β-glucosidase activity can be detected in the upper epidermis, stratum granulosum and stratum corneum [33]. In a similar prodrug approach to that adopted here, dermal β- glucocerebrosidase activity was used to generate δ-tocopherol and retinoic acid when these compounds were delivered as glycosides [34].

A range of skin commensal bacteria and common dermal pathogens were tested for the ability to hydrolyse esculin to esculetin. Swabs taken from human skin and a number of strains commonly found on the skin generated detectable esculetin within 24−48 h of incubation, including *S. aureus* strains NCTC10442, NCTC6571, NCTC10788, ATCC25923 and DSMZ11729, *C. albicans* SC5314, *E. faecalis* ATCC29212, and *K. pneumoniae* ATCC8849. Under these conditions, two strains of *S. epidermidis* (ATCC35984 and ATCC12228) did not hydrolyse esculin. Irrespective of esculin hydrolysis, all microbes tested were able to grow on medium containing esculin, indicating that 1 g/l esculin, and any esculetin produced, were not antibacterial. This concentration is significantly higher than esculetin concentrations sufficient to kill dermatophytes. It would seem that hydrolysis of esculin to esculetin, and therefore generation of the active “drug” may not be dependent on the action of dermatophytes alone. If insufficient β–glucosidase were present on the skin, irrespective of source, an alternative approach would be to create a topical agent that contains both esculin and a β–glucosidase, possibly together in the same cream or separately in a dual tube dispenser that mixes them on application. Amyloglucosidase derived from *Rhizopus niveus* for use in degrading gelatinized starch into constituent sugars is found in the FDA Generally Recognized as Safe (GRAS) and alpphaglucosidase alpha is used clinically for the treatment of Pompe disease, so a precedent for such a use of glucosidases has already been set.

### Conclusions

Our data suggests that selected water-soluble coumarin glycosides have potential efficacy in the treatment of common superficial mycoses, based on the ability of common dermatophyte pathogens and autochthonous members of the skin microbiota to produce β-glucosidases capable of hydrolysing these “pro-drugs” and releasing the antifungal aglycone. It is interesting to note that a plant defence mechanism based on β-glucosidase activity and plant glycones has been proposed by Yamada and co-workers. In this system, glycones (including the coumarin glycone scopolin) accumulate in the cell vacuole and β-glucosidases accumulate in ER-bodies. The aglycone of scopolin, scopoletin (7-hydroxy-6-methoxycoumarin) has antifungal activity and is closely related to fraxetin (7,8-dihydroxy-6-methoxycoumarin). When the cells are damaged, either by herbivore predation or pathogen attack (e. g. *Pseudomonas syringae* [35], the β-glucosidases and glycones are mixed, hydrolysing the glycones to aglycones that can mount a defence for the plant [36]. A next stage to the development of esculin as a topical treatment for superficial mycoses will be in vivo testing using a rodent model of a superficial mycosis.

## Materials and Methods

### Test organisms, coumarins and other chemicals

*Trichophyton interdigitale* NCPF0335 (human skin) and *T. rubrum* NCPF0118 (human nail) were obtained from NCPF (HPA, UK). Clinical isolates from tinea patients, were supplied by Professor Michel Monod (Univ Lausanne, Switzerland). Fungi were grown on Potato Dextrose agar (Oxoid Ltd, UK) at 30°C for ≤14 d.

Esculin sesquihydrate [6-(β-D-glucopyranosyloxy)-7-hydroxycoumarin.1.5 H_2_0] was supplied by Pharmasynthese SAS (France). Esculin [6-(β-D-glucopyranosyloxy)-7-hydroxycoumarin], Esculetin [6,7-dihydroxycoumarin], Daphnetin [7,8-dihydroxycoumarin] and Fraxetin [7,8-dihydroxy-6-methoxycoumarin] and all other chemicals were obtained from Sigma-Aldrich (UK).

Initial studies focused on the solubility of the coumarins and coumarin glycosides for use in this study, following determination of their theoretical partition coefficients (logP). The coumarin glycosides, esculin and fraxin were more soluble in RPMI-1640 medium and water, ≥6.806 and 7406.2 mg/ml, respectively) than the corresponding aglycones esculetin and fraxetin 0.871 and 1.041 mg/ml) and this correlated with lower (negative) logP values of the glycosides (−0.53 and 1.38 for esculin and esculetin, respectively, and −0.66 and 1.74 for fraxin and fraxetin, respectively). Solubilisation of coumarin aglycones in aqueous solutions was only possible following prior dissolution in 100% (v/v) DMSO. The maximum DMSO concentration used experimentally, <1% (w/v), was not toxic to tested fungi.

### Antifungal susceptibility testing

Antifungal susceptibility testing to determine the Minimum Inhibitory Concentration (MIC) was conducted using the broth micro-dilution procedure [37]. The MIC at which no growth was observed (MIC_100_) was determined by changes in absorbance (530 nm) for up to 7 d at 30°C (PowerWave Microplate Spectrophotometer, BioTek Instruments Inc, USA) by comparison with relevant controls. In all cases, standard deviations were within 10% of the mean. The minimum fungicidal concentration (MFC) was determined following MIC experiments for wells in which no growth was observed [38]. All experiments were conducted in triplicate on triplicate samples.

### Determination of β-glucosidase activity

Replicate cultures of *T. rubrum* NCPF0118 and *T. interdigitale* NCPF0335 were grown in 10 ml RPMI-1640 liquid medium and incubated statically at 30°C for 10 weeks. At set time-points, triplicate samples were removed and assayed for intracellular and extracellular β-glucosidase activity.

β-glucosidase activity was determined using a modification of the method of Workman and Day [39], scaled down to a microplate format. In brief, an aliquot of the sample to be analysed was mixed with Phosphate/Citrate buffer and exposed to 2 mM 4-nitrophenyl-β-D-glucopyranoside and incubated for 30 min at 30°C. The reaction was stopped by the addition of 0.1 mM NaOH and absorbance at 405 nm was measured. β-glucosidase activity was quantified by comparison with an almond β-glucosidase (Sigma-Aldrich, UK) standard curve. For the determination of specific enzyme activity, protein concentrations were determined using the Pierce BCA Protein Assay (ThermoFisher, UK) according to the manufacturer's instructions (microplate procedure). Samples containing phenol red were treated with the Pierce Compat-Able Protein Assay Kit (ThermoFisher, UK) prior to the protein assay.

### Effect of conditioned supernatants on antifungal activity

To assess the effect of conditioned supernatants on the antifungal effect of esculin the antifungal susceptibility testing was conducted with the exception that 40% (v/v) of the final volume of each well contained 8 week old conditioned supernatant from the same strain of *Trichophyton* spp. Inocula were prepared in 1.2 x RPMI-1640 to ensure that the final medium concentration and inoculum size was the same as described above.

### Effect of the β–glucosidase inhibitor conduritol β-epoxide (CBE) on antifungal activity

Inhibition of *Trichophyton* spp. β–glucosidase activity was carried out the using irreversible inhibitor Conduritol β–epoxide (CBE) [26] at a final concentration of 40 µg/ml, followed by monitoring growth for up to 168 h at 30°C.

### Hydrolysis of esculin by bacteria typical of the skin microbiota

The basis of this assay was the bile-esculin assay [40], with the omission of bile. Esculin (1 g/l) and ferric ammonium citrate (0.5 g/l) were added to slopes of the appropriate growth medium; Mueller-Hinton (MH) agar (bacteria; 37°C), Sabouraud Dextrose (SabDex) agar (yeast; 30°C) modified Leeming-Notman (Malassezia spp.; 30°C), spread with the relevant inoculum and incubated for up to 48 h. Analysis of the skin microbiota was assessed by sampling from the groin of a volunteer. Skin samples were incubated on MH and SabDex. Esculin hydrolysis was indicated by the formation of a dark brown/black complex in the medium following the reaction between esculetin and ferric ammonium citrate.
